# Determination of H-Atom Positions in Organic Crystal Structures by NEXAFS Combined with Density Functional Theory: a Study of Two-Component Systems Containing Isonicotinamide

**DOI:** 10.1021/acs.jpca.2c00439

**Published:** 2022-05-10

**Authors:** Paul T. Edwards, Lucy K. Saunders, David C. Grinter, Pilar Ferrer, Georg Held, Elizabeth J. Shotton, Sven L. M. Schroeder

**Affiliations:** † School of Chemical and Process Engineering, University of Leeds, Leeds LS2 9JT, U.K.; ‡ Diamond Light Source, Harwell Science & Innovation Campus, Didcot OX11 0DE, U.K.; § Future Continuous Manufacturing and Advanced Crystallisation Hub, Research Complex at Harwell (RCaH), Rutherford Appleton Laboratory, Didcot OX11 0FA, U.K.

## Abstract

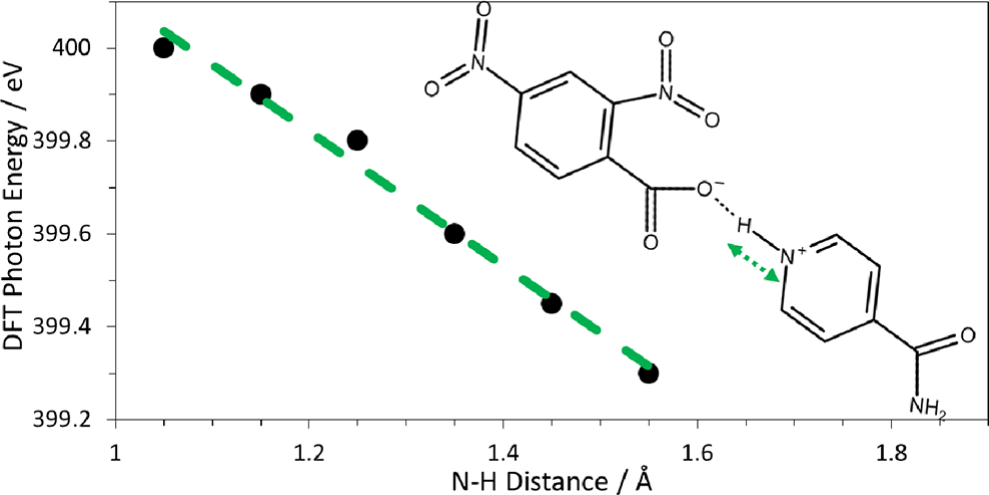

It is important to be able to identify the precise position of H-atoms in hydrogen bonding interactions to fully understand the effects on the structure and properties of organic crystals. Using a combination of near-edge X-ray absorption fine structure (NEXAFS) spectroscopy and density functional theory (DFT) quantum chemistry calculations, we demonstrate the sensitivity of core-level X-ray spectroscopy to the precise H-atom position within a donor-proton-acceptor system. Exploiting this sensitivity, we then combine the predictive power of DFT with the experimental NEXAFS, confirming the H-atom position identified using single-crystal X-ray diffraction (XRD) techniques more easily than using other H-atom sensitive techniques, such as neutron diffraction. This proof of principle experiment confirms the H-atom positions in structures obtained from XRD, providing evidence for the potential use of NEXAFS as a more accurate and easier method of locating H-atoms within organic crystals.

## Introduction

**Figure 1:**
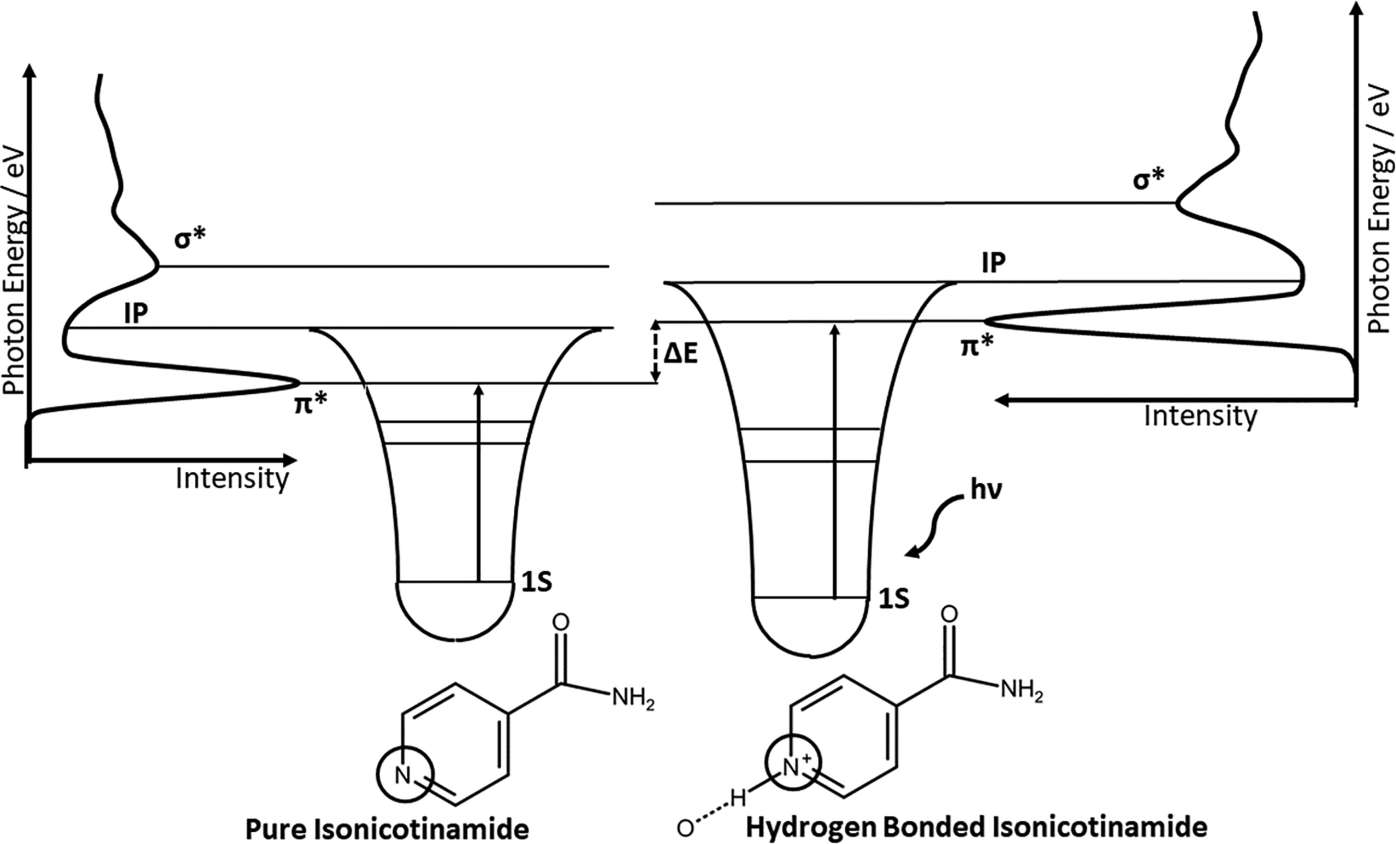
Schematic of the principal transitions for the pyridinium nitrogen showing the energy levels of the 1s, π*, and σ* orbitals and the ionization potential. The effect of hydrogen bonding and proton transfer increasing the photon energy of the N 1s → π* transition is shown due to stabilizing of the N 1s orbital in the hydrogen-bonded complex.

Hydrogen bonding and Brønsted proton transfer interactions are fundamental to the formation of organic crystal structures.^1^ The location of the H-atom between the donor and acceptor atoms in these interactions has an important effect on the structural properties of the crystal; understanding is particularly relevant for regulatory bodies identifying structures for patent protection of pharmaceutical compounds.^2−4^ Hydrogen bonding in cocrystals and proton transfer in salts form the extremities of the salt-cocrystal continuum describing the two states where the hydrogen atom is either located close to the proton donor (cocrystal) or the proton acceptor (salt).^1,4,5^ Our previous studies have consistently demonstrated how core-level spectroscopy in the form of X-ray photoelectron spectroscopy (XPS) is sensitive to the position of the H-atom through the 1s core-level binding energy (BE) shift at the proton acceptor, as shown in Figure 1.^3,6−9^ This effect is also present in near-edge X-ray absorption fine structure (NEXAFS) spectroscopy through a shift dependent upon both the core-level BE and the energy of the final state unoccupied molecular orbitals.^3,7−17^ Due to the large number of unoccupied π* (where π bonding is present) and σ* orbitals, which are both highly sensitive to the molecular and electronic structures,^17^ the technique has the potential to enable a structure refinement directly sensitive to the H-atom. The origin of the features in a NEXAFS spectrum are outlined in Figure 1, with the energy of the N 1s → π* transition indicated by the length of the arrow. The shift in energy from the pure isonicotinamide to the hydrogen-bonded complex can be attributed to the effect of the complex lowering the energy of the 1s orbital, resulting in a shift in the photon energy of Δ*E*.

Previous studies have focused on the direct effects on the experimental NEXAFS spectra for identifying bond lengths. The so-called “bond length with a ruler” method has been proposed^12,17−20^ identifying trends between the energy position of the ionization potential and σ* shape resonances. In many cases, this provides a useful tool to identify differences between similar compounds. However, the approach is not sufficiently sensitive to conformational variations and other noncovalent interactions to act as a more general structure refinement tool.^12^

Complementing experimental NEXAFS, time-dependent density functional theory (TDDFT) calculations can be conducted to model the theoretical NEXAFS spectrum based upon the calculated excitation energies and transition dipole moments.^21^ The approximations made in the formalism of DFT have been shown to not adversely affect the properties calculated.^21−23^ One of the most common exchange correlation functionals used for DFT calculations is the B3LYP functional,^24^ and there have been studies where this is shown to underestimate excitation energies compared to the experiment.^22,23,25^ However, relative excitation energies are found to be correct, and therefore a uniform shift is generally applied to allow comparison to experimental data.^11,26^ Using this approach, it is possible to model the effect of H-atom position on the experimental NEXAFS spectrum, with the aim of identifying how sensitive the technique is to the precise location of the H-atom.

An alternative commonly used method for finding such a precise H-atom location is neutron diffraction, although sample preparation is more onerous and data acquisition is more laborious.^27,28^ The difficulty of locating H-atoms using X-ray diffraction (XRD) stems from the fundamental insensitivity of the technique to H-atom positions. The diffraction of X-rays relies on electron density at the scattering sites, and by their nature, hydrogen atoms have low electron density.^29^ Since this electron is occupied in a bond, this electron density is also biased toward the donor atoms to which the H-atom is bonded.^27^ This leads to a general lack of sensitivity to the exact H-atom location and a requirement for additional refinement methods for H-atom positions.^30,31^ Here, we aim to complement and improve on this theoretical refinement modeling with NEXAFS spectroscopy and DFT calculations exploring the determination of the location of H-atoms within a hydrogen bond for systems for which their location has previously been determined.

In a recent study, we examined three isonicotinamide systems using XPS, in which isonicotinamide forms 1:1 salts with 2,4-dinitrobenzoic acid and 3,5-dinitrobenzoic acid and a 2:1 structure with phthalic acid containing two distinct interactions between the components: one Brønsted proton transfer interaction and one standard hydrogen bond.^6,31^ For these isonicotinamide 2,4-dinitrobenzoic acid (IN24DNBA), isonicotinamide 3,5-dinitrobenzoic acid (IN35DNBA). and isonicotinamide phthalic acid (INPA) systems, the expected chemical shift of the N 1s BE was observed, confirming that the core-level BEs are consistent with the crystallographically determined interactions. Using NEXAFS, we have now investigated these complexes further to confirm the XRD-based H-atom location procedure experimentally (the XRD-derived hydrogen bond distances can be found in Table S1 of the Supporting Information).^31^ In effect, this forms the basis of a “proof of principle” experiment showing how NEXAFS could be used to complement structure refinement by XRD.

## Modeling and Experimental Methods

### DFT Calculations

DFT calculations were completed using the ORCA software package version 5.0.1, utilizing the University of Leeds high performance computing facilities.^32,33^ Example ORCA input file and xyz files for calculations are available in the Supporting Information. For molecular geometry optimization calculations, the B3LYP^24^ exchange correlation functional was used along with the def2-TZVP basis set,^34^ def2/J auxiliary basis set, and the RIJCOSX approximation.^35^ For the ground-state geometry optimization, this is a sufficient level of theory allowing accurate molecular geometries to be calculated in reasonable timescales. Additionally, geometry optimization of larger clusters of molecules was calculated using the same approximations. Starting structures were obtained from the Cambridge structural database (CSD).^36^ Geometry optimization parameters were all left at the default values in ORCA. Hydrogen position optimization was carried out on all structures (such that the other atoms remained in the XRD measured positions), in addition to a full optimization of every atom position in the molecule. Where a full optimization was done, a vibrational frequency calculation was also completed to ensure the structure reached a global minimum.

For the modeling of NEXAFS spectra, TDDFT calculations were run on various crystal and DFT-optimized structures. TDDFT is used to calculate the excited states and can be used to model an X-ray absorption spectrum based on the transition probabilities between occupied and unoccupied molecular orbitals and the energies calculated.^21^ By allowing only transitions from the core-level orbitals of specific atoms, we can calculate a theoretical NEXAFS spectrum accounting for all of the electronic transitions within a certain energy range.^21^ The same B3LYP^24^ exchange correlation functional and def2-TZVP basis set were used in all calculations.^34^ By default, the Tamm-Dancoff approximation^37^ was applied which simplifies the calculation of excited states without significantly affecting the energies calculated and improving them for some cases involving triplet excited states. Due to the consistent underestimate of excitation energies using this exchange correlation functional, a uniform shift of typically +12.4 eV was applied to all calculated spectra.

### Materials

All three samples were prepared by evaporation from solution, as described in previous work.^31^ The crystallizations were carried out in methanol (INPA) and ethanol (IN24DNBA and IN35DNBA) using a 2:1 stoichiometric ratio (INPA) or 1:1 ratio (IN24DNBA and IN35DNBA).^31^ The individual components for the crystallizations [isonicotinamide (99.9%), 2,4-dinitrobenzoic acid (98%), 3,5-dinitrobenzoic acid (99%), and phthalic acid (99.5%)] were obtained from Sigma-Aldrich.

### NEXAFS

NEXAFS characterization of the powder samples was carried out at Diamond Light Source beamline VerSoX B07-B utilizing the near ambient pressure NEXAFS end station enabling fast sample transfer.^38^ Measurements were carried out using total electron yield detection, measuring the drain current through the sample mounting plate with the soft X-ray beam at normal incidence and at room temperature. Samples were mounted on metal sample holders with carbon tape on a copper foil, with the crystalline powders pressed onto the carbon tape. The photon energy scale was calibrated for beamline effects and surface charging using a small trace of nitrogen gas in the sample chamber which is therefore present in the gas-phase spectra. This is well known to form a peak at 400.8 eV,^39,40^ to which the photon energy scale was calibrated by applying a uniform shift to each spectrum. The sample chamber was operated with He gas at 1 mbar. He was chosen as there are no absorption peaks in the region around the C, N, and O K-edges and because it minimizes the electron yield signal of fluorescence gas phase absorption. The incident X-ray beam intensity *I*_0_ is required for determining the absorption spectrum (see the Supporting Information) and was acquired by measuring the He gas-phase spectrum when the sample plate was removed from the beam path. There is a difference in the gas-phase beam path from which electrons are emitted when the sample is removed in this way, and this needs to be accounted for during the spectrum normalization process (see Supporting Information). Athena XAS analysis software^41^ was used to model the spectra through curve fitting, with error functions to model the absorption edge steps associated with ionization at the photoemission thresholds and Gaussian bell curves to model the absorption lines associated with core excitations to unoccupied bound states.

## Results

DFT modeled spectra were calculated using the orca_mapspc program within the ORCA software suite^33^ using a Gaussian line broadening of 0.6 eV to compute the X-ray absorption spectra as this best matches the experimental spectra for the N 1s → π* transitions. The photon energy scale is shifted by approximately 12 eV such that the main peak matches with the experimentally observed value. This is necessary due to the consistent underestimation of excitation energies calculated using the B3LYP exchange correlation functional.^22^ An alternative exchange correlation functional, designed for the calculation of core-level properties,^42^ was tested as well; see Figure S5. While the absolute values were generally in agreement with the experiment, the relative offset of the peak positions compared poorly to the experiment, particularly for the amide groups. We also calculated the spectra using a range of larger basis sets (including additional polarization functions and additional diffuse functions) to investigate the basis set convergence. We observed that additional diffuse basis functions lower the energies of the outermost orbitals, reducing the excitation energies of electrons into these molecular orbitals. However, the peaks of interest, in particular, the N 1s → π* transitions, remained at the same energy, leading to the decision to use the lower computational cost of the smaller def2-TZVP basis set.^34^ The calculations using alternative exchange correlation functional and basis sets are available in the Supporting Information (Figures S4 and S5).

### Isonicotinamide 2,4-Dinitrobenzoic Acid (IN24DNBA)

**Figure 2:**
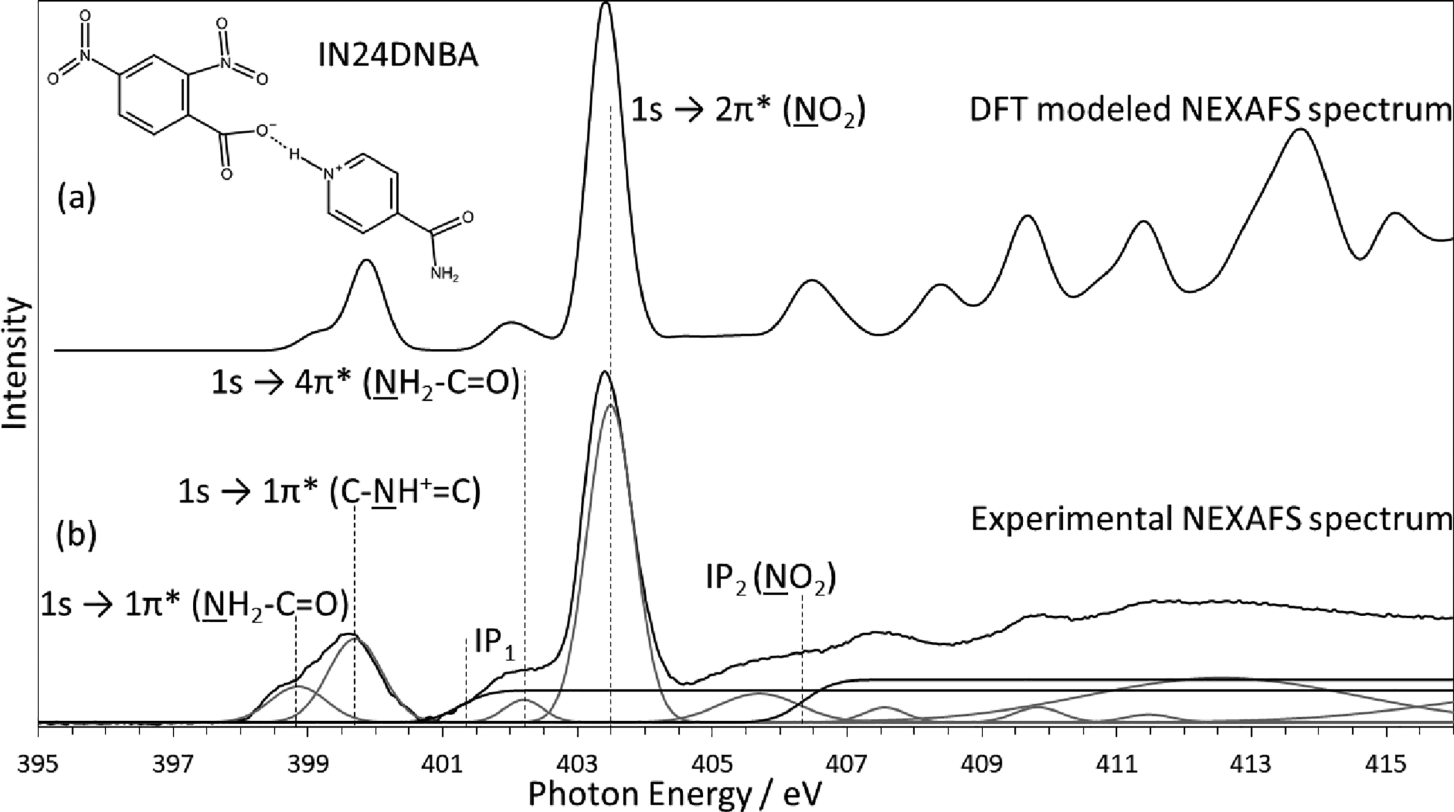
Modeled and experimental N K-edge NEXAFS spectrum of IN24DNBA showing excellent agreement of π* resonance peak positions. Experimental NEXAFS spectrum is fitted with Gaussian peaks identifying transitions and error functions at the core electron binding energies determined using XPS. N 1s → π* transitions are identified based on the origin of the core-level electron and the π* orbital related to the relevant ring structure. Gaussian peaks at photon energies above 405 eV are to allow data fitting only, and we extract no physical meaning from these.

According to X-ray crystallography, the IN24DNBA complex forms a 1:1 salt structure.^31^ Using XPS, we confirmed this through an N 1s BE shift at the proton acceptor of +2 eV compared to a cocrystal, with relative N 1s emission intensities for all nitrogen moieties in line with the bulk stoichiometry, confirming that the composition within the near-surface region probed by XPS is the same as the bulk.^6^Figure 2 shows the modeled and experimental nitrogen K-edge NEXAFS spectra for this salt. It is immediately obvious that the modeled structure very closely resembles the experimental NEXAFS data. The photon energy scale of the modeled spectrum was shifted by 12.15 eV from the originally calculated value using the nitro group 1s → 2π* transition for photon energy scale calibration. Peak assignments are aided through the use of DFT modeling since the electronic transitions responsible for each peak can be directly identified, as shown in Figure 2b. The three nitrogen environments [amide (NH_2_–C=O), pyridinium ring (C–NH^+^=C), and nitro (−NO_2_)] are each associated with a distinct N 1s → π* resonance, consistent with our previous XPS analysis.^6^ The nitro group is the most intense and visible at a photon energy of 403.5 eV. The protonated nitrogen of the pyridinium results in the peak at 399.7 eV, while the amide 1s → 1π* transition appears as a low-energy shoulder to the protonated nitrogen, the peak of that shoulder being at 398.9 eV. Figure 2b shows the experimental spectrum including Gaussian peak fitting and step functions for the ionization potentials using the N 1s binding energies determined from XPS. Higher-energy peaks in the electron backscattering regime are also fitted with Gaussians as this facilitates more accurate fits in the NEXAFS range, but we stress that Gaussians are physically meaningless in this region, which reflects oscillatory extended X-ray absorption fine structure variations of the absorption coefficient. The low-energy shoulder to the nitro peak at 402.2 eV can easily be attributed to the amide N 1s → 4π* transitions, while higher-energy transitions are primarily due to transitions to σ* orbitals (shape resonances). The intensities of the transitions associated with the three nitrogen species are influenced by the nature of the initial and final states. This is unlike XPS, where the peak area is proportional to the excitation cross section of the atomic initial state and therefore proportional to the concentration of the excited moiety within the probed volume. The absorption intensity in NEXAFS is additionally influenced by the net transition rate from the excited core-level to the unoccupied molecular orbitals, resulting in the relatively weak amide absorption band. In previous work, we have not observed this effect to the same extent,^12,13,19,43^ but encouragingly, the DFT calculated spectrum predicts the same relative intensities, confirming this analysis.

### Isonicotinamide 3,5-Dinitrobenzoic Acid (IN35DNBA)

**Figure 3:**
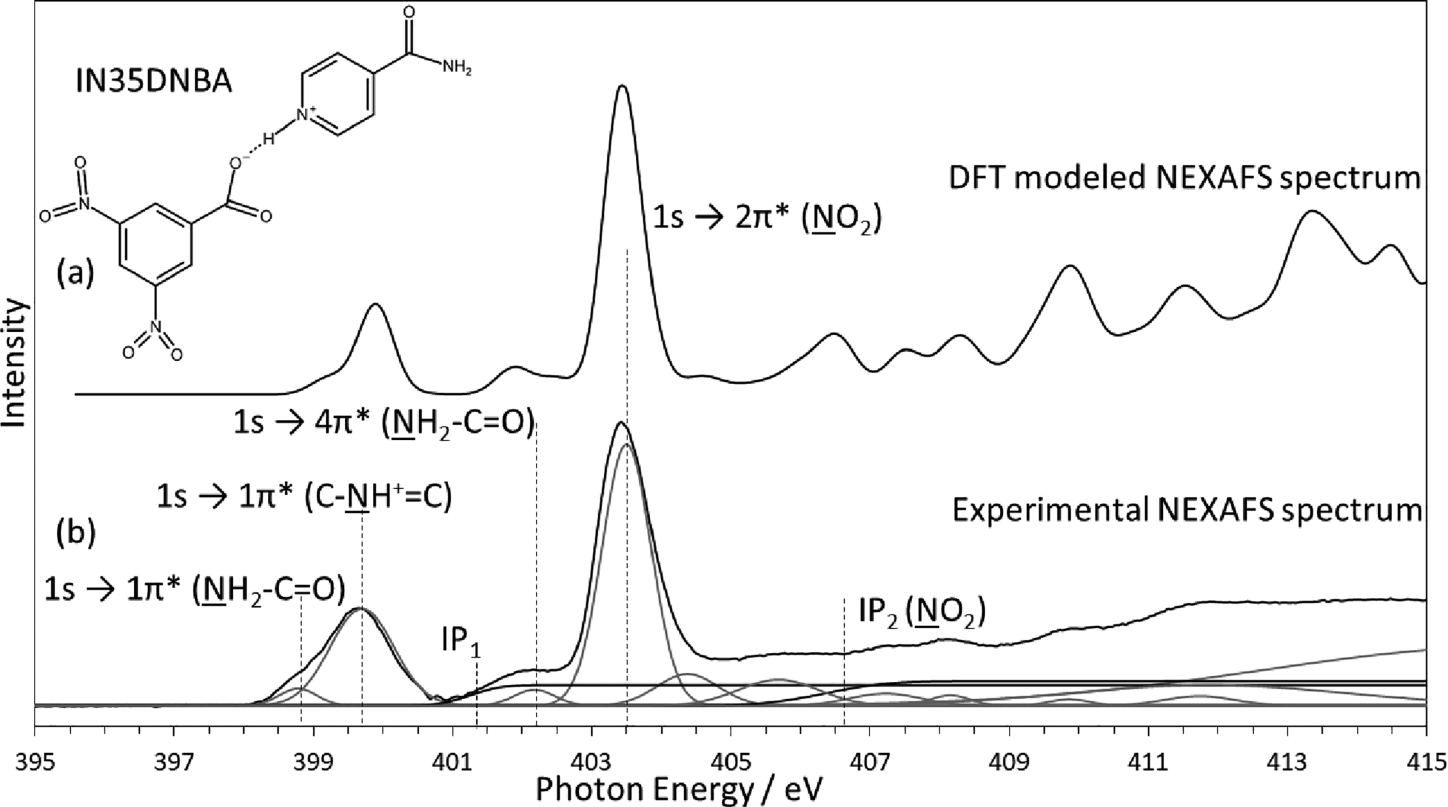
Modeled and experimental N K-edge NEXAFS spectrum of IN35DNBA showing excellent agreement of π* resonance peak positions. Overall peak shapes also fully consistent with the salt structure with a low-energy amide peak (398.8 eV) and higher-energy protonated nitrogen peak (399.7 eV). Additional feature compared with IN24DNBA acid above the nitro peak (404.4 eV) present in both spectra visible as a slight broadening.

The IN35DNBA complex forms a 1:1 salt structure.^31^Figure 3 shows the experimental and modeled NEXAFS spectra for this sample. It is immediately clear that the overall peak shapes form the same pattern as IN24DNBA, with an additional shoulder peak above the nitro peak, and different σ* resonances, again with the modeled spectrum suggesting larger intensities than observed experimentally. This is primarily due to a much larger width of the σ* resonances in the experimental spectrum leading to lower maximum intensities. The differences between the spectrum for IN24DNBA and IN35DNBA demonstrate the sensitivity of the NEXAFS technique compared with other core-level spectroscopies. When these samples were measured using XPS, the resultant spectra are identical, and only the slight differences in the unoccupied molecular orbitals allow us to distinguish between these samples using NEXAFS as we know the core-level BE is essentially the same. This is an important result, indicating just how sensitive NEXAFS is to the chemical environment and highlighting the importance of the entire NEXAFS region of XAS spectra, not only the most prominent π* resonances. Table 1 summarizes the photon energies of the N 1s → π* transitions. We clearly observe that the energies for these transitions are almost identical between IN24DNBA and IN35DNBA—in agreement with XPS. It is the additional information and the higher-energy σ* resonances in the spectra which allow us to distinguish between the two similar complexes using NEXAFS. For the majority of the σ* resonances in both IN24DNBA and IN35DNBA, there are corresponding peaks in both the experimental and modeled spectra, suggesting that the difference between the complexes is real. The fit for IN35DNBA follows the same principles as IN24DNBA and is shown in Figure 3b. The peak intensity for the amide component forms a smaller proportion of the peak areas than for IN24DNBA. This could be due to the slightly different molecular orientations in this complex and the differing positions of the nitro groups. Additionally, the peak fitted just above the nitro group 1s → 2π* fits with the slight additional shoulder peak observed theoretically in the DFT calculated spectra. The fit is consistent with previous work on the sample, including XRD and XPS studies.

**Table 1:** Best Fit Photon Energies (eV) for the N 1s → π* Transitions^a^

		N–C=O	C–N=C	C–NH=C	–NO_2_
IN24DNBA	energy/eV	398.9	n/a	399.7	403.5
	DFT energy/eV	399.2		399.9	403.5
IN35DNBA	energy/eV	398.8	n/a	399.7	403.5
	DFT energy/eV	399.2		399.9	403.5
INPA	energy/eV	398.8	398.8	399.7	n/a
	DFT energy/eV	399.5	398.8	399.6	
	DFT energy^b^/eV	398.9	398.8	399.6	
	DFT energy^c^/eV	398.8	398.7	399.6	

a DFT energies after uniform shift applied taken from the calculated excitation energies.

b Additional changes to the molecular structure to reproduce peak shifts and intensities from the experimental spectrum.

c Molecular cluster calculation to account for longer-range interactions.

### Isonicotinamide Phthalic Acid (INPA)

**Figure 4:**
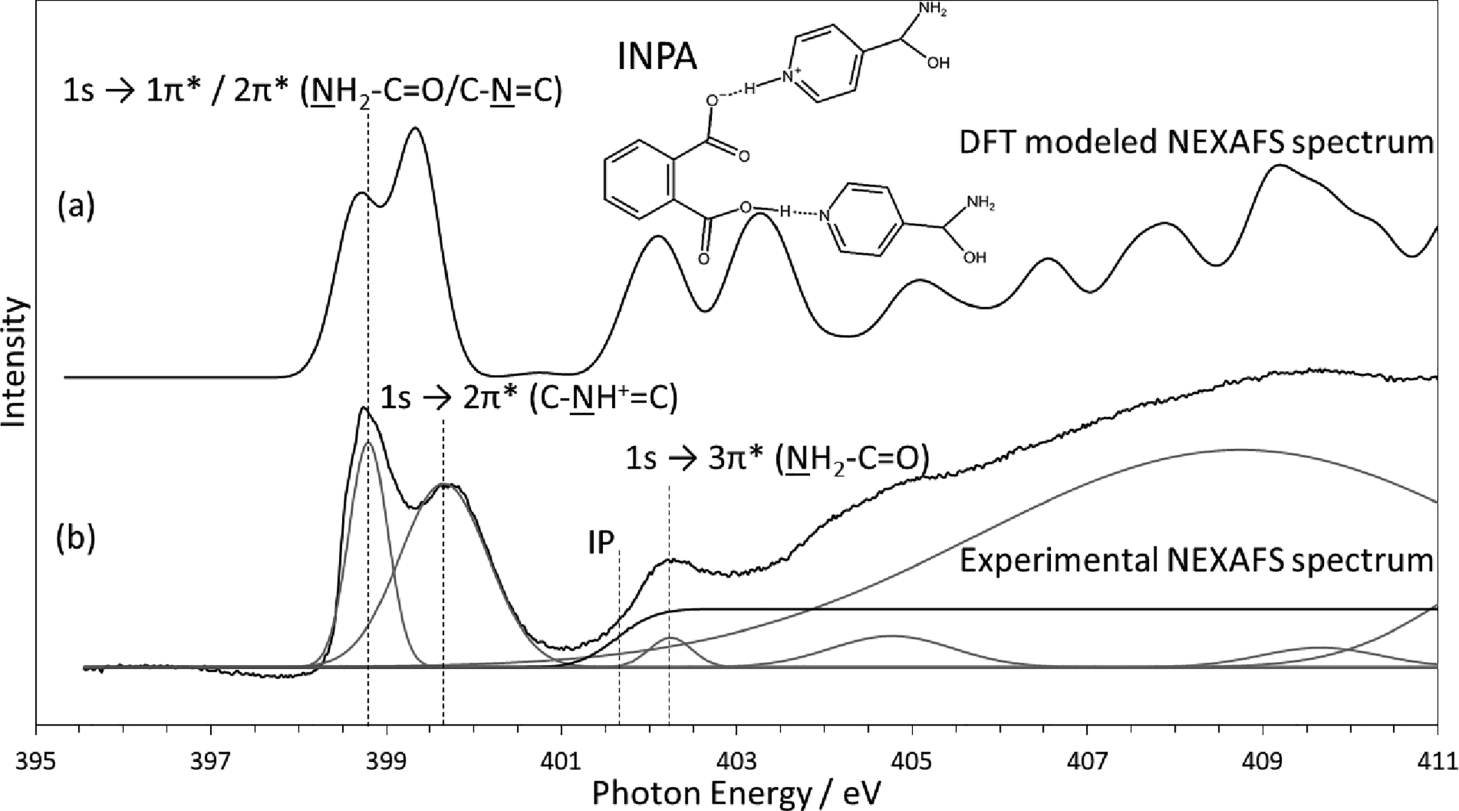
Modeled and experimental N K-edge NEXAFS spectrum of INPA showing excellent agreement of π* resonance peak positions. However, the DFT calculated spectrum has altered peak intensities for the peaks at 398.8 and 399.7 eV, suggesting a difference in the structure.

The INPA complex forms a 2:1 structure containing one hydrogen bond interaction and one proton transfer interaction between the phthalic acid and isonicotinamide components.^31^ In XPS measurements, this structure exhibits a shift of +1.7 eV in the N 1s BE related to one of the two nitrogen acceptors, confirming this analysis.^6^ The experimental NEXAFS spectrum shown in Figure 4b appears to confirm this structure, with an obvious increase in the photon energy of the protonated nitrogen acceptor N 1s → 2π* peak, leading to the distinctive double peak. In contrast to the other two complexes, the DFT-calculated NEXAFS spectrum (Figure 4a) shows some differences in the relative intensities of the peaks and an additional feature at 403.3 eV which is absent in the experimental spectrum. Using DFT modeling, we were able to assign the π* resonances to transitions from the core levels of each of the nitrogen atoms in the structure. Figure 4b shows our assignment of the N 1s → π* transitions based on knowledge of the XP spectra and relative intensities of the peaks observed. We have already seen that the interaction cross section for the amide groups is significantly lower than for the pyridine ring nitrogen. Therefore, both the amide and unprotonated pyridine ring nitrogen energies overlap, leading to the additional intensity in the lower energy peak at 398.8 eV. This photon energy is in excellent agreement with the other two complexes, as shown in Table 1. Interestingly, the higher-energy protonated nitrogen N 1s → 2π* peak at 399.7 eV exhibits broadening compared to the lower peak. There is no reason for this based on the instrumentation used, so this feature may suggest the presence of disorder in the H-atom position, potentially in agreement with previous work.^31^ Our analysis using the DFT calculation indicates that the energy of the amide nitrogen shifts to contribute to the higher of the two peaks, explaining the difference in relative intensities observed. These NH_2_ groups form weak hydrogen bonds to oxygen at neighboring amide groups and carboxylic acid groups in phthalic acid. Additionally, unlike the other two complexes, the higher-energy σ* peaks do not align well with the experimental data, with several peaks aligning with troughs. It is likely that longer-range interactions between multiple components influence the energies of the amide N 1s → π* and σ* peaks which are not reproduced in calculations involving only an isolated complex.

## Discussion

### Isonicotinamide Phthalic Acid Peak Intensities

**Figure 5:**
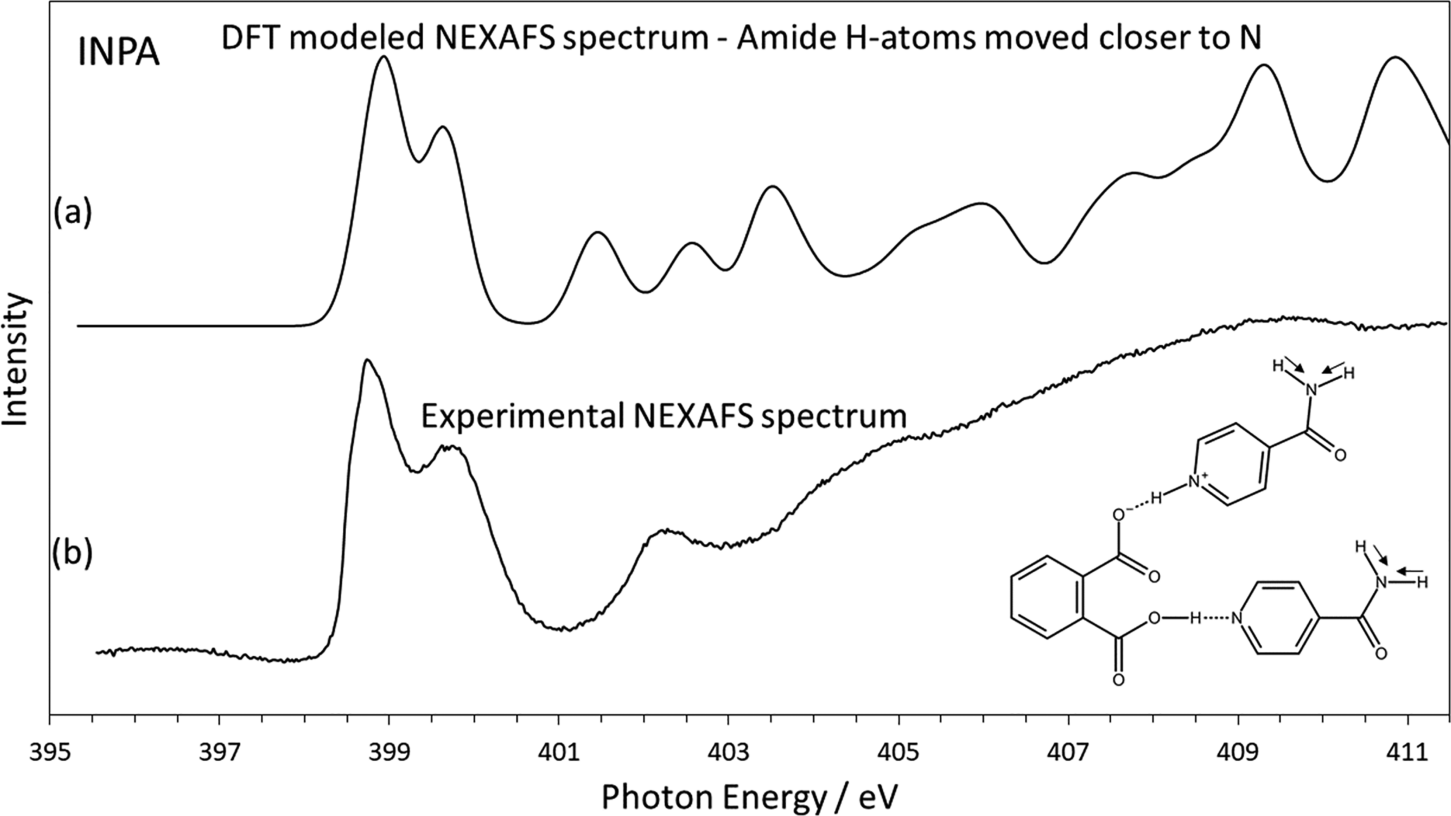
Modeled and experimental N K-edge NEXAFS spectrum of INPA showing excellent agreement of π* resonance peak positions and peak intensities. N–H distances at the amide groups have been reduced by 0.15 Å compared to the original database geometry to correctly match the experimental spectrum.

To assess the sensitivity of NEXAFS to the H-atom location we have carried out a calculation scanning across a range of N–H distances at the amide nitrogen to investigate the effect on the NEXAFS spectrum of INPA. By moving these H-atoms closer to the amide nitrogen by 0.15 Å, while leaving all other atomic positions as reported in the CSD untouched, we find that the energy of the 1s → π* resonance decreases to that observed experimentally (Figure 5 and Table 1). Figure 5 shows this modeled spectrum compared to the experimental spectrum, and the relative intensities of the double peak appear to be corrected compared to the CSD geometry (Figure 4). This result is a demonstration of the high sensitivity of NEXAFS to the H-atom location. It also underlines our idea of a refinement procedure combining NEXAFS with DFT to locate H-atoms more accurately within hydrogen bonds. The simplistic approach taken here, movement of the H-atom while leaving the remaining structure static, is not meant to imply that the best fit H-atom location is a correct representation of the crystal structure. Indeed, the resulting N–H distance of 0.77 Å is too short to be realistic. However, it does give insight into the sensitivity of the NEXAFS technique, with a 0.15 Å change in the N–H distance leading to a chemical shift of −0.7 eV.

### DFT of Cluster Structures—INPA

**Figure 6:**
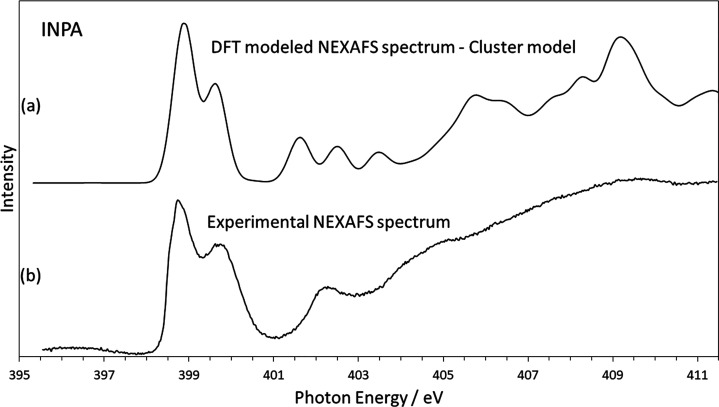
Modeled and experimental N K-edge NEXAFS spectrum of INPA showing excellent agreement of π* resonance peak positions and peak intensities. Calculation based on a cluster of molecules to simulate longer-range interactions involved in the crystal structure.

We have also calculated the INPA spectrum using a larger cluster of molecules, at the CSD geometry, to investigate the effects of longer-range interactions on the predicted spectrum. The details for IN24DNBA and IN35DNBA are shown in Figure S2 in the Supporting Information as the longer-range interactions have much less of an effect in these complexes. For INPA, the importance of longer-range interactions on the energy of the amide nitrogen resonances has become clear, with Figure 6 showing the cluster model spectrum compared with the experimental data. The relative peak intensities are predicted correctly in this case; the only difference between this and the initial calculation being additional molecules in the cluster. To avoid any effect of the peripheral molecules in the structure, the theoretical spectrum was formed based on transitions from the central molecule only. This improvement in the energy of the amide peak shows how vital a correct molecular geometry is when calculating a theoretical spectrum. The σ* resonances are slightly improved over the initial calculation, with the higher-energy peaks aligning with the experimental data and the unexplained peak at 403.3 eV being significantly reduced in intensity. Another effect not visible in the DFT calculation is the broadening of the protonated nitrogen peak. This further suggests the broadening is due to some disorder of the H-atom location, with the DFT calculation focusing on just one conformation. To simulate this effect, an average over a range of H-atom positions (at the protonated nitrogen site) has to be taken.

### The Effect of H-atom Position on Peak Position

**Figure 7:**
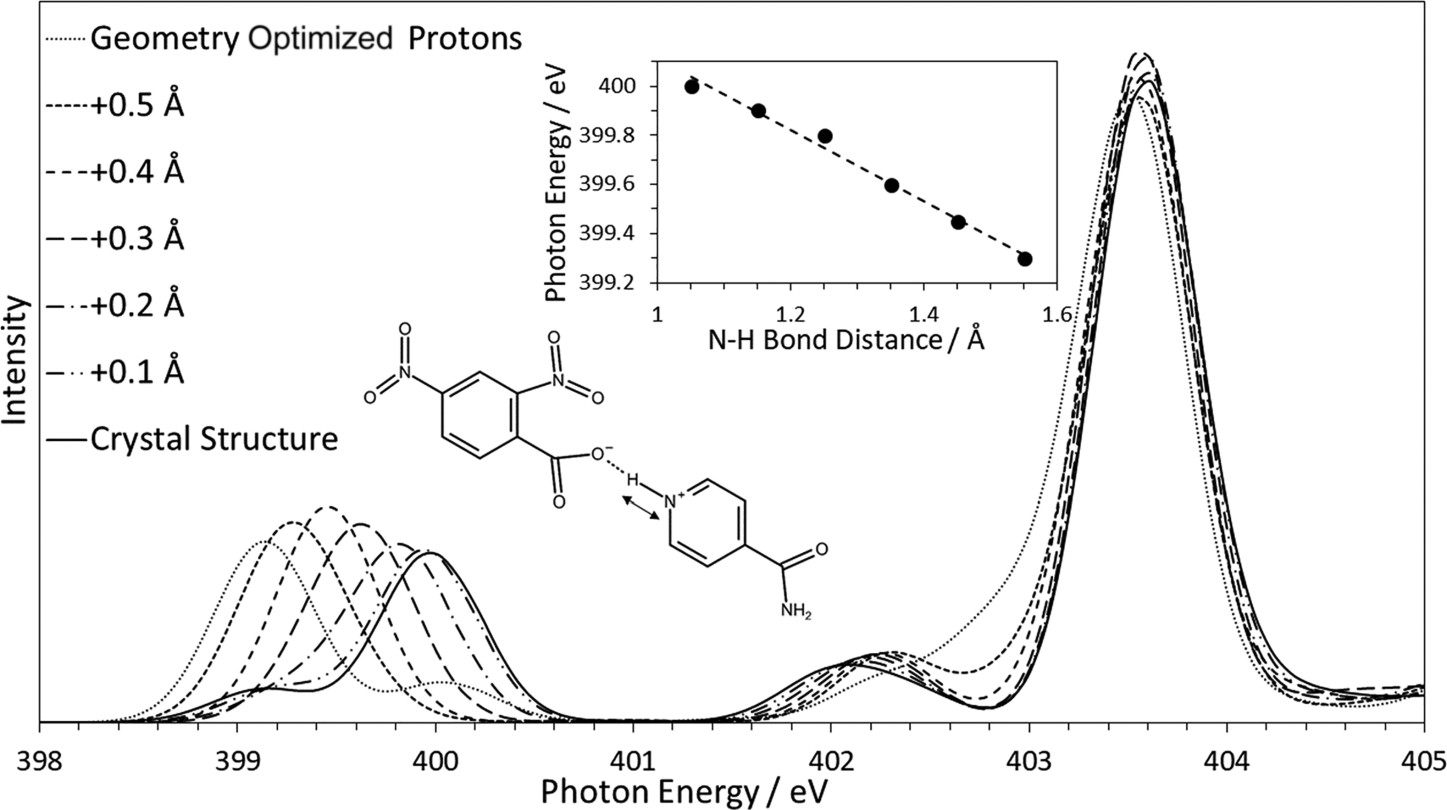
Series of DFT-calculated NEXAFS spectra moving the H-atom across the hydrogen bond from the structure in the crystal database to the “optimized hydrogen” structure. The peak positions for the amide nitrogen are different for the “optimized hydrogen” structure calculation since all hydrogen atom positions were optimized.

In order to quantify how sensitive the spectra are to the location of the H-atom, an important test was to observe the theoretical effect of moving the H-atom on the calculated NEXAFS spectrum. An initial test of this was to calculate the spectra for the three samples for a range of N–H distances from the CSD structure to the optimized hydrogen structure in 0.1 Å steps, changing no other parameters of the geometry. Figure 7 shows the effect on the IN24DNBA nitrogen K-edge spectrum. As expected, both the nitro peak (403.5 eV) and the amide peaks (399.2 and 402.2 eV) remain in the same position (except for the optimized hydrogen structure where all H-atom positions are changed). We observe a continuous shift in the photon energy calculated for the nitrogen acceptor, showing that, at least theoretically, the core-level excitation energies are sufficiently sensitive to the H-atom location to identify a difference of 0.1 Å. The inset plot in Figure 7 shows the relationship between the photon energy calculated and the N–H bond distance. Using this roughly linear relationship, the bond length can be determined based on the experimental photon energy. Further to this, the CSD structure shows excellent agreement with the experimental spectrum (see Figure 2), including relative intensities and peak positions, indicating that the H-atom location is accurately refined in the XRD analysis in these relatively simple samples. This theoretical approach implies a direct sensitivity of the nitrogen acceptor π* resonance energy to the position of the H-atom and opens up a new way to crystal structure refinement utilizing this rapid and simple technique.

### Difference Spectra-Complexes Compared to Individual Components

**Figure 8:**
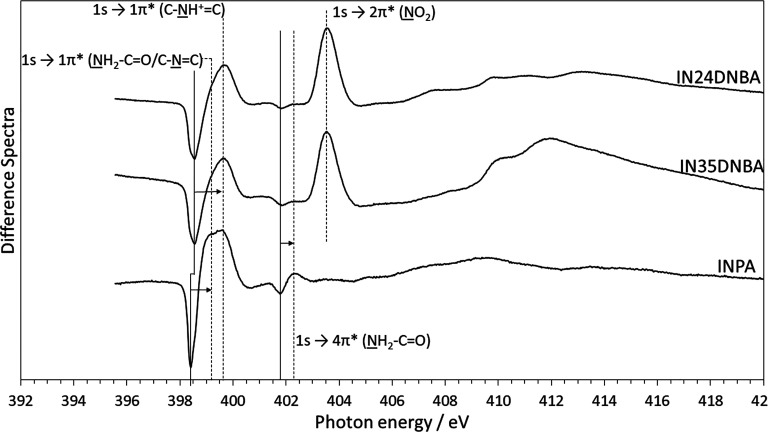
Difference spectra between experimental spectra and sum of the individual component’s experimental spectra (see Figure S3). All three complexes exhibit a clear shift of both the amide and pyridine ring nitrogen species from the pure components to the crystal complex. Negative peaks (solid lines) correspond to the peak position in the pure compounds, and arrows indicate the peak shift from pure compound to crystal complex.

To visualize the effect of hydrogen bonding and proton transfer interactions on the spectrum, difference spectra for the three complexes were calculated using experimental data for the individual components of the complexes. The difference spectra between the individual components and the complexes were calculated by subtracting the sum of the components in the correct stoichiometric ratio from the experimental spectrum of the complex. This is shown in Figure 8. Care was taken to normalize each of the spectra with respect to the high-energy baseline (which in effect is proportional to the N 1s XPS intensity) to ensure the relative signals from each of the components were correct. Here, we can clearly observe the N 1s → 1π* peaks shift from an energy of 398.5 eV in pure isonicotinamide to higher energy in the crystal complex and a broadening of the peak as a result of the splitting of the peak into the amide (399.2 eV) and pyridinium (399.7 eV) nitrogens. This is particularly obvious in INPA because of the double peak due to the additional unprotonated pyridine nitrogen, leading to an even more pronounced broadening in the difference spectrum. The trough (401.8 eV) and peak (402.3 eV) corresponding to the amide N 1s → 4π* transition shows an equivalent increase in energy due to the forming of the complex. The N 1s → 2π* peak at 403.5 eV is due to the measured intensity being higher in the complexes than the pure dinitrobenzoic acid components, even with full normalization of the spectra. We also observe some additional features at higher photon energies due to changes in the unoccupied molecular orbitals when the complex is formed.

We have previously shown that the core-level BE of electrons (determined using XPS) is directly affected by the distance between the H-atom and the proton acceptor.^6^ However, the magnitude of the BE shifts also depends on the surround electrons and atoms, and therefore there is no universal dependency.^6^ This means we have reached the limit of usefulness of XPS in the characterization of organic hydrogen bonded crystals. The use of NEXAFS utilizes the increased sensitivity and structural dependence of the spectra on both the core N 1s level and the unoccupied π* orbitals to determine more precisely the location of the H-atom within the hydrogen bond by combining the predictive power of DFT with NEXAFS.

## Conclusions

The combination of experimental and theoretical nitrogen K-edge NEXAFS spectra has been used to accurately determine the structure of three two-component organic crystals by optimizing the H-atom locations and comparing to H-atom location refinement procedures used in XRD. The observed chemical shifts and relative intensities of nitrogen acceptor atoms are used to combine the predictions of DFT calculations with the NEXAFS experiment to obtain a fully consistent picture of the crystal structure and the important interactions. For all three complexes, the chemical shift and relative intensities calculated for the protonated nitrogen match exactly with experiment based on the CSD molecular geometries, indicating that the XRD proton location refinement in these structures is correct. In INPA, this peak is broadened, suggesting some degree of disorder as mentioned in a previous XRD-based paper.^31^ Ignoring longer-range interactions between neighboring molecular units leads to incorrect peak positioning of amide nitrogen N 1s → 1π* resonances in DFT calculations, indicating the importance of these interactions on the observed crystal structure. In addition, the combination of DFT with NEXAFS for refinement of H-atom positions has been established through a theoretical demonstration of the effect of H-atom position on the measured spectra, with changes in the peak position above the detection limit for changes in the H-atom position of <0.1 Å. The advantage of NEXAFS over XRD is its fundamental direct sensitivity to the H-atom locations. This approach is also more practical than alternatives such as neutron diffraction, with measurements of the nitrogen K-edge typically taking on the order of 15 min, allowing rapid sample analysis. We intend to use this technique to refine the H-atom location in some more challenging situations, particularly where there is uncertainty in the H-atom location within a hydrogen bonded system, such as quasi-centered short strong hydrogen bonds.

## Supporting Information Available

Chemical structure and hydrogen bonding information, experimental NEXAFS normalization procedure, additional fitted spectra and fitting parameters, cluster model for IN24DNBA and IN35DNBA and spectra of individual components, and DFT calculation input files and geometry files with structural data, including comparison of exchange correlation functional and basis sets (PDF)
