# Supplementary material for: Determination of H-Atom Positions in Organic Crystal Structures by NEXAFS Combined with Density Functional Theory: a Study of Two-Component Systems Containing Isonicotinamide
Source: J Phys Chem A. 2022 May 10;126(19):2889–98. doi: 10.1021/acs.jpca.2c00439 (PMC9125558; doi:10.1021/acs.jpca.2c00439)
Supplement: Supplementary file 1 — jp2c00439_si_001.pdf [file jp2c00439_si_001.pdf]

Determination of H-atom Positions in Organic Crystal Structures by NEXAFS Combined with Density Functional Theory: A study of Two-Component Systems Containing Isonicotinamide – Supporting information

Paul T. Edwards,[1, 2] Lucy K. Saunders,[2] David C. Grinter,[2] Pilar Ferrer,[2] Georg Held,[2] Elizabeth J. Shotton,[2] Sven L. M. Schroeder[1,2,3]**

[1]School of Chemical and Process Engineering, University of Leeds, Leeds, LS2 9JT, UK
[2]Diamond Light Source, Harwell Science & Innovation Campus, Didcot, OX11 0DE, UK
[3]Future Continuous Manufacturing and Advanced Crystallisation Hub, Research Complex at Harwell (RCaH), Rutherford Appleton Laboratory, Didcot, OX11 0FA, UK

* Sven L. M. Schroeder:

Email: s.l.m.schroeder@leeds.ac.uk

* Elizabeth J. Shotton:

Email: elizabeth.shotton@diamond.ac.uk

The three crystal structures investigated including hydrogen bonding information and hydrogen bond distances

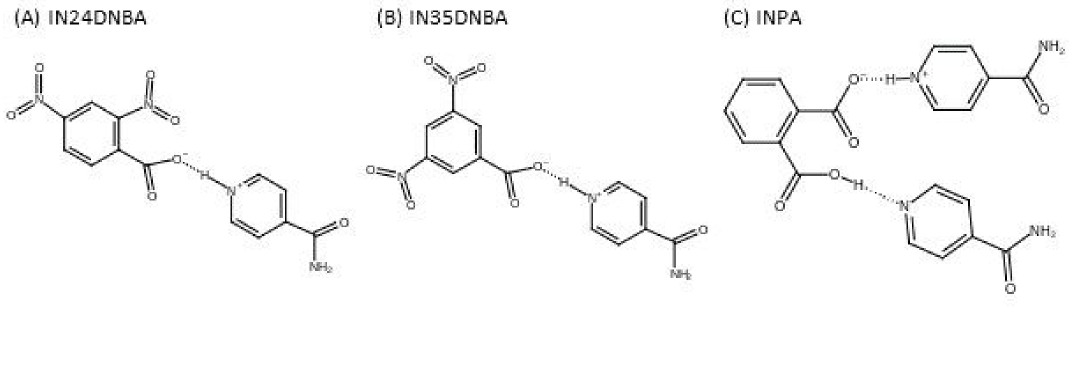

Figure S1. Chemical structures for the three systems investigated: (A) isonicotinamide 2,4-dinitrobenzoic acid, (B) isonicotinamide 3,5-dinitrobenzoic acid and (C) isonicotinamide phthalic acid including the relevant donor-proton-acceptor interactions present in the structure.

The three two-component organic crystals studied in this work are: isonicotinamide 2,4-dinitrobenzoic acid ($C_{13}H_{10}N_4O_7$), isonicotinamide 3,5-diniotrobenzoic acid ($C_{13}H_{10}N_4O_7$) and isonicotinamide phthalic acid ($C_{20}H_{18}N_2O_6$). The chemical structures are shown in Figure S1 including all of the relevant intermolecular hydrogen bonding and proton transfer interactions. These interactions have previously been characterized using X-ray diffraction (XRD), and the bond distance and angle parameters for each interaction are shown in Table S1.[1]

Table S1. Hydrogen bond and proton transfer interaction bond distances and angles from XRD for the three systems.

	N–H / Å	H···O / Å	N···O / Å	N–H···O / °
IN24DNBA N–H···O	1.046	1.572	2.600	166.2
IN35DNBA N–H···O	1.084	1.484	2.535	161.4
INPA				
N–H···O	1.102	1.449	2.550	176.7
N···H–O	1.662	1.042	2.696	179.2

NEXAFS normalization procedure for NAP-NEXAFS using the gas phase (with longer path length) as the background I_0

Due to the difficulties of measuring the background signal when using NAP-NEXAFS, measurements of the He gas phase (the chamber with sample removed from the beam path) were taken to provide a background. Significant features are present in this spectrum at the nitrogen K-edge due to the use of a silicon nitride membrane in the beamline.

The absorption coefficient (intensity of a NEXAFS spectrum) is defined by μ, where I is the measured spectrum and I_0 is the background spectrum:

$$\mu = \frac{I}{I_0}$$

By using NAP-NEXAFS, the measurement of the I_0 background becomes non-trivial, with the effect of the gas phase important in the normalization of the measured spectra. To measure an approximate I_0, a gas phase measurement (by removing the sample plate from the beam path) was taken at each of the required absorption edges. Because of the difference in path length through the gas phase compared

to the measurements of samples, this approximate I_0 is offset by a value n and scaled by a factor m, resulting in I_0^*.

$$I_0^* = mI_0 + n$$

Through a series of rearrangements, we can then obtain the true absorption spectrum, divided by a constant m, which is dependent upon a single variable n, which is fit to best model the spectrum baseline.

$$\frac{\mu}{m} = \frac{I}{I_0^*}\left(\frac{1}{1 - \frac{n}{I_0^*}}\right)$$

The value of n should be a spectrometer and condition dependent constant, and results in the standard equation where the value is 0 (i.e. no offset). Therefore, to identify the appropriate value of n, we suggest minimizing the value off n while retaining a baseline with physical meaning (i.e. no negative obtrusions). We found a value of n=0.005 appropriate for our experiments.

Fitted spectra – peak parameters

Fitting parameters have been determined using the Athena XAS analysis software[2] to fit Gaussian peaks to the NEXAFS spectra. These fits, shown in Table S2 are consistent with our previous XPS study[3] and the relative intensities align well with the calculated DFT spectra.

Table S2. Best fit photon energies (eV), Relative intensities and Gaussian peak width for the N 1s → π* transitions. *single peak used to describe transitions from two nitrogen environments

N1s→π* transition		<u>N</u>–C=O	C–<u>N</u>=C	C–<u>N</u>H$^+$=C	–<u>N</u>O$_2$
IN24DNBA	Energy / eV	398.9	n/a	399.7	403.5
	Relative intensity	0.348		0.673	2.746
	Width (σ)	0.35		0.35	0.36
IN35DNBA	Energy / eV	398.8	n/a	399.7	403.5
	Relative intensity	0.111		1.073	2.231
	Width (σ)	0.27		0.46	0.35
INPA	Energy / eV	398.8	398.8	399.7	n/a
	Relative intensity	0.430*	0.430*	0.798	
	Width (σ)	0.22*	0.22*	0.51	

Isonicotinamide 2,4-dinitrobenzoic acid & Isonicotinamide 3,5-dinitrobenzoic acid cluster calculations

In addition to the INPA calculations on a larger cluster of molecules, the equivalent calculations were completed on the other two systems. The overall result was consistent with the INPA, with a similar decrease in energy of the amide groups. However, in these cases, this shift was larger than observed in the experimental spectrum, as seen in Figure S2. This amide nitrogen is highly affected by the longer range interactions, and in these cases the DFT calculation overestimates the relaxation effect of the additional interactions in the system leading to the shift indicated by the arrows in Figure S2.

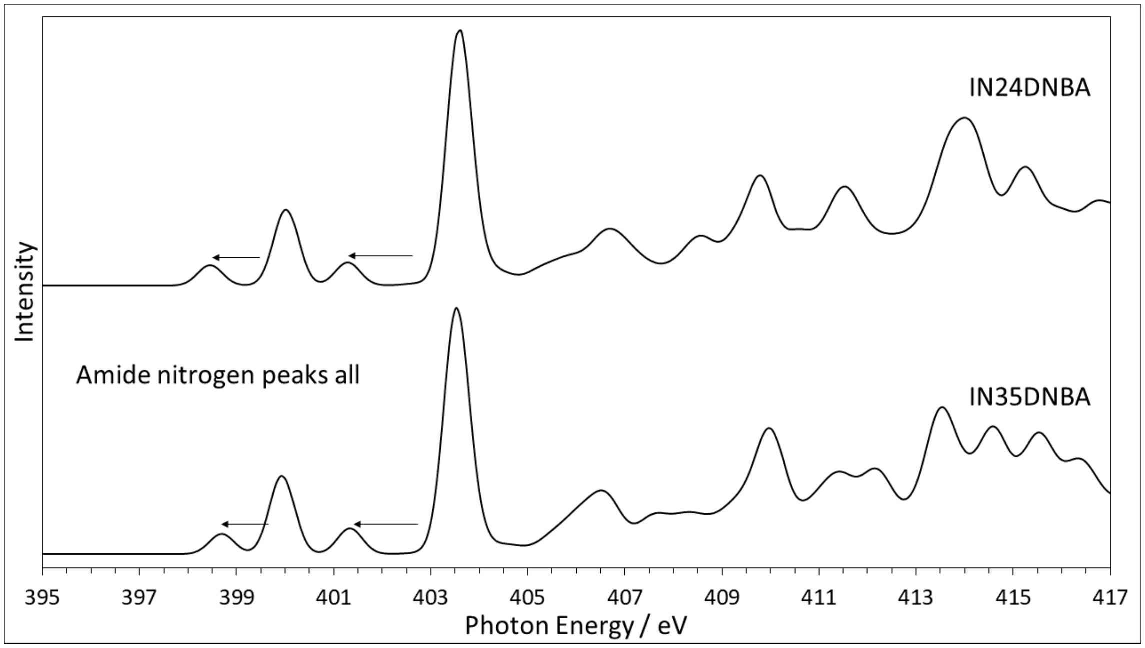

Figure S2. DFT Calculated spectra based on a cluster of molecules to observe the effect of longer range interactions on the spectra. It is clear that the energy of the amide peaks (shown with arrows), rather than the pyridine (399.9 eV) or nitro (403.5 eV), are affected by the longer range interactions

Crystal component spectra

The Individual components of the three crystals were also measured to determine the difference spectra between the sum of the components and the formed crystal. These are shown in Figure S3. Isonicotinamide has three clear peaks corresponding to the transitions from the core level N 1s orbitals to the $1\pi^*$, $2\pi^*$ and $3\pi^*$ orbitals respectively. In pure isonicotinamide, the core level of both the pyridine ring and amide nitrogen are at the same energy (as observed in XPS measurement) and therefore both contribute to each peak.

The spectra of 35DNBA and 24DNBA are very similar, with slightly different σ* resonances. The spectrum of 24DNBA also has an additional feature at 400 eV, although this is anticipated to be some form of contamination, as there should not be a feature at this energy. Importantly, this small peak does not appear to affect the difference spectra as seen in the manuscript.

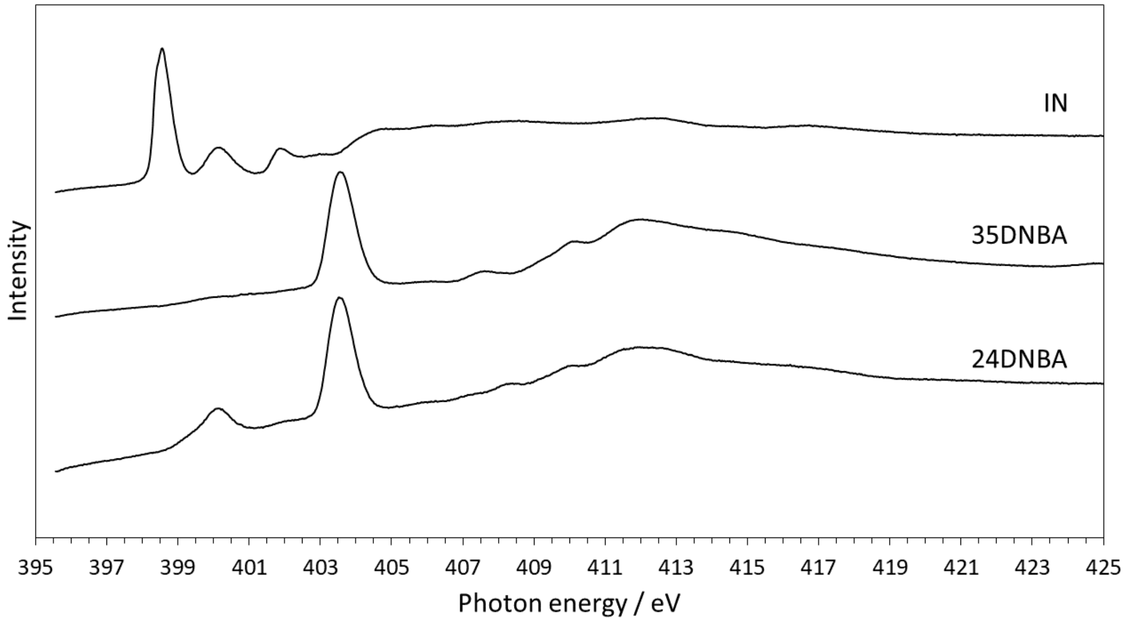

Figure S3. Experimental NEXAFS spectra of the pure components of the systems. Used to determine the difference spectra. Note: Phthalic acid does not contain nitrogen so does not contribute directly to the N K-edge spectrum.

Density Functional Theory Calculation Details

Example input files and the xyz files for the calculations are included here for reference purposes. *.xyz files were obtained from the CSD based on XRD structure refinement. Most TDDFT calculations were done directly using these structures, but some were also carried out after an optimization calculation of the hydrogens in the structure.

Example input file for a hydrogen atom only constrained geometry optimization

```
! B3LYP def2-TZVP def2/J RIJCOSX opt freq

%geom
optimizehydrogens true
end

* xyzfile 0 1 in35dnba.xyz
```

Example input file for a TDDFT excited state calculation

```
! B3LYP def2-TZVP def2/J RIJCOSX

%tddft
orbwin[0]=7,10,-1,-1
doquad true
nroots 800
end

* xyzfile 0 1 in35dnba_opt.xyz
```

*.xyz files for the three complexes from XRD studies.[1]

48			
Isonicotinamide Phthalic Acid			
H	7.615970	9.656497	7.321674
H	7.315375	8.071444	3.343656
H	7.548208	10.003317	4.855522
H	7.508525	7.340543	8.211975
H	7.314089	3.954033	8.466086
O	6.060540	4.877835	3.928695
O	7.146430	4.932295	8.127826
O	7.757451	4.055500	6.152620
O	8.110379	5.229528	3.103202
C	7.407626	6.403413	6.292070
C	7.202081	5.472445	3.909463
C	7.452692	5.012980	6.840144
C	7.495985	9.009148	5.258077
C	7.359272	6.606489	4.899763
C	7.481025	7.507791	7.150013
C	7.533677	8.805069	6.638318
C	7.396943	7.909930	4.398123
H	7.894837	0.298093	11.610475
H	7.256790	2.667074	10.990738
H	8.074566	2.036492	7.020032
H	9.043512	-2.275826	8.020021
H	8.656249	-0.358285	7.420393
H	9.236816	-3.468199	9.314340
O	8.569074	-1.954516	11.143844
N	7.647250	2.425733	8.985276
N	9.001606	-2.516451	8.993319
C	8.644537	-1.645818	9.947422
C	7.935156	0.649786	10.569719
C	8.323041	-0.226866	9.555836
C	7.593482	1.957383	10.239558
C	8.378546	0.256245	8.248904
C	8.045077	1.589352	8.015085
H	6.107399	0.478669	0.488112
H	6.336583	1.901777	5.164477
H	6.066225	2.892077	1.188287
H	6.412641	-0.561791	4.648943
H	5.735133	-3.538423	2.665418
H	5.287425	-2.293024	3.806174
H	6.131878	3.542722	3.477110
O	6.669769	-1.971857	0.974066
N	5.795765	-2.569047	2.984976
N	6.184137	2.439061	3.189727
C	6.245115	-1.679210	2.101439
C	6.145669	0.744660	1.531427
C	6.225676	-0.229302	2.526115
C	6.304006	0.167391	3.863943
C	6.127950	2.079486	1.898334
C	6.276818	1.523141	4.165768

34			
Isonicotinamide 2,4 Dinitrobenzoic Acid			
H	3.797843	3.469588	4.716098
H	5.107453	7.042507	3.678873
H	2.176963	3.523550	2.941065
H	0.846284	6.484525	-0.290378
H	2.244801	6.985101	0.279079
H	3.522494	7.241130	1.884632
H	5.276526	5.260640	5.003087
O	0.669273	4.583025	1.307604
N	1.604780	6.400598	0.321675
N	4.540562	5.279354	4.259855
C	2.691414	5.384406	2.244271
C	1.572953	5.431249	1.232355
C	4.497605	6.358463	3.471541
C	3.582994	6.439749	2.437705
C	3.709143	4.234689	4.075346
C	2.767271	4.264884	3.068854
H	8.346000	4.903578	7.747553
H	6.943716	1.145814	10.130460
H	9.549559	4.192898	9.658172
O	6.116094	5.050651	6.315437
O	6.000592	2.913674	5.665306
O	4.374058	2.110341	7.859411
O	10.265007	2.615165	11.365187
O	8.641616	1.288524	11.922102
O	5.517797	0.271758	8.083578
N	5.405267	1.497250	8.109452
C	6.296811	3.812529	6.478816
N	9.152252	2.125152	11.187797
C	8.383075	2.573144	10.013856
C	6.964781	3.405409	7.778399
C	8.069418	4.124585	8.231705
C	6.592426	2.273258	8.504343
C	7.269652	1.838354	9.634105
C	8.801301	3.709658	9.338869

34			
Isonicotinamide 3,5 Dinitrobenzoic Acid			
O	3.807306	1.827214	-0.045287
N	4.765867	0.392795	1.428422
N	6.132029	4.986923	2.971947
C	4.510099	1.612532	0.955636
C	5.307673	3.976291	0.998407
C	5.108689	2.785911	1.692490
C	5.949276	3.852386	3.662571
C	5.836801	5.067785	1.669217
C	5.444619	2.721047	3.048054
H	5.327050	1.906723	3.593592
H	6.002327	5.932174	1.243291
H	5.280926	0.250876	2.196201
H	5.138745	4.060263	0.083864
H	6.167620	3.880510	4.586338
H	4.375508	-0.343477	0.992746
H	6.493154	5.783573	3.501342
O	9.097109	12.227690	8.412026
O	6.485519	8.714984	3.933034
O	9.807369	7.770221	10.112587
O	9.086823	6.065025	8.978006
O	7.046218	6.659588	4.645777
O	7.621909	12.525933	6.848792
N	9.247943	7.268552	9.138397
N	8.331047	11.811668	7.556398
C	8.726815	8.175216	8.103614
C	8.131130	7.623617	6.976161
C	8.831648	9.540269	8.321766
C	8.250354	10.355272	7.360155
C	7.614807	9.860762	6.232283
C	7.580326	8.481048	6.024614
C	6.969659	7.920054	4.753753
H	7.225718	10.448573	5.587470
H	9.291378	9.945743	9.087764
H	8.112045	6.693352	6.858017

Effect of Basis set and Exchange Correlation functional on the calculated NEXAFS spectra

As mentioned in the main manuscript, to ensure an appropriate basis set and exchange correlation

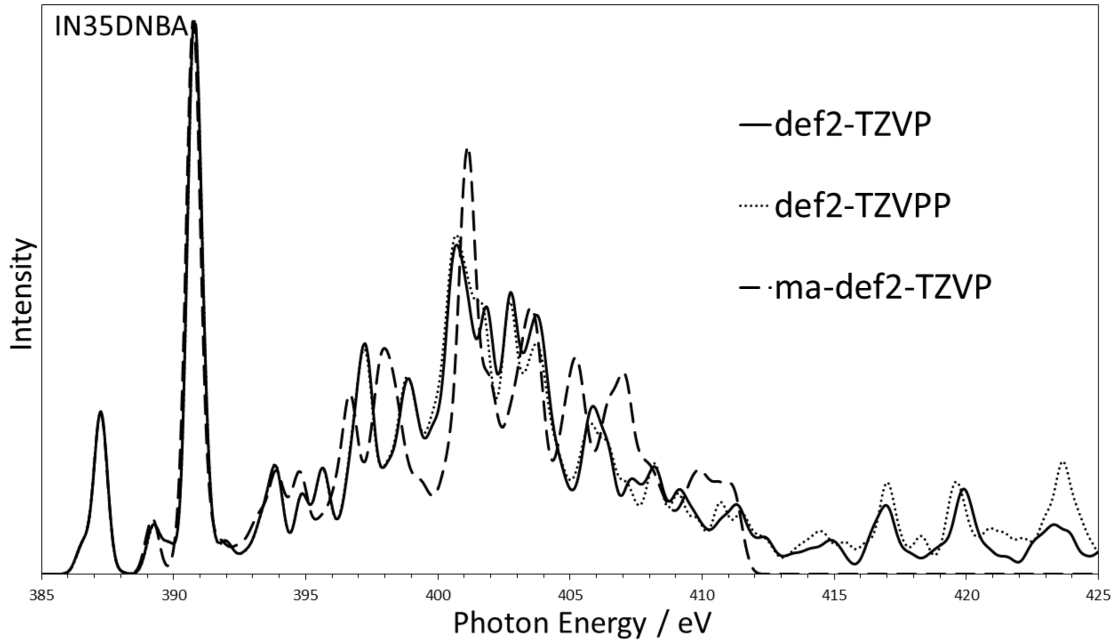

Figure S4. DFT calculated NEXAFS spectra demonstrating the effect of basis set on the N 1s → π* transitions and σ* resonances. Photon energies have not been calibrated to allow for direct comparison of the calculated spectra. The primary N 1s → π* peaks are unaffected by the change in basis set, hence the decision to utilize the smaller def2-TZVP basis set for the calculations completed for this work.

functional were utilized in the calculations, a series of tests were completed to ensure the basis set convergence was reasonable (at least in the region of the spectrum of interest) and the exchange correlation functional gave reasonable results. Figure S4 shows the calculated NEXAFS spectrum of IN35DNBA using three different basis sets. The standard triple zeta valence polarized, with additional polarization functions, and augmented with additional basis functions. The important feature to note is that the basis set does not affect the position or intensity of the main N 1s → π* transitions.

The two exchange correlation functionals tested were B3LYP and SRC1 (Utilizing the error function based range separation method mixing BLYP and Hartree Fock exchange components). These calculations were done on the "crystal structure" from the database, so no geometry optimization was required, and therefore it is the NEXAFS spectrum calculation (TDDFT) that we are probing. Figure S5 shows the effect of the two functionals, with B3LYP reproducing a spectrum resembling

experimental data, while SRC1 does not require any photon energy scale calibration. However, the relative peak positions are off compared to experiment and therefore the B3LYP functional was used.

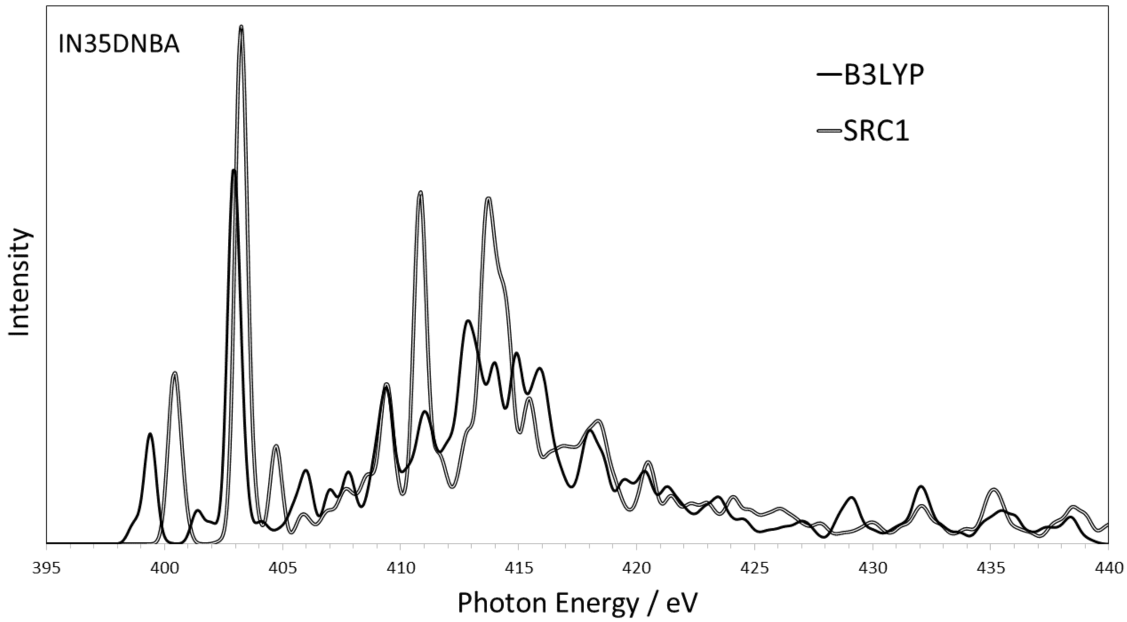

Figure S5. DFT calculated NEXAFS spectra demonstrating the effect of exchange correlation functional on the N 1s → π* transitions and σ* resonances. The B3LYP Photon energies have been calibrated to the Nitro peak, the SRC1 functional Photon energies have not been calibrated since the calculated values are close to experiment. Relative peak positions are more consistent with experiment for the B3LYP calculation.

Effect of Exchange Correlation Functional on Geometry Optimization

For completeness, we also include here the test of the two exchange correlation functionals for geometry optimization. Having optimized the geometry using the two separate functionals, Figure S6 shows the Calculated NEXAFS spectra, using the B3LYP functional for the TDDFT calculation as decided previously. Both exchange correlation functionals result in almost indistinguishable NEXAFS spectra, with only a few very minor differences in peak intensity between the two optimized structures. Therefore, since the choice of functional for geometry optimization had an insignificant effect on the calculated NEXAFS spectra, we used the same B3LYP functional for all optimizations and TDDFT calculations for consistency and reduced computational cost.

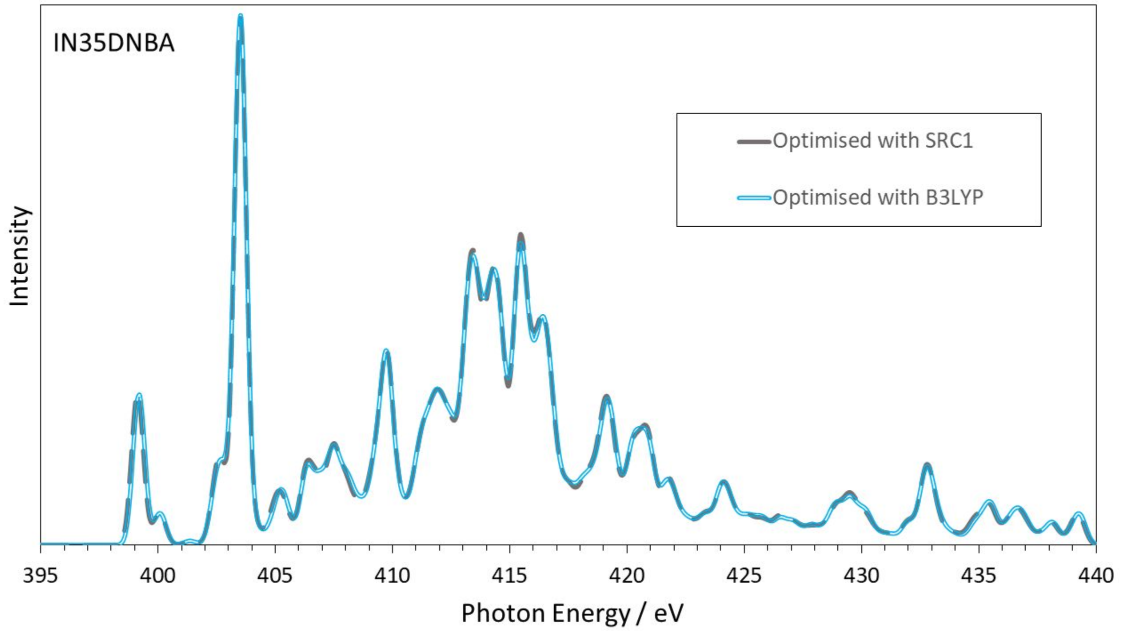

Figure S6. DFT calculated NEXAFS spectra demonstrating the effect of exchange correlation functional on the geometry optimization process. Both spectra are almost indistinguishable with only minor changes in peak intensity present. TDDFT calculated using B3LYP functional in both cases, as this is shown to be more consistent with experiment, see Figure S5.

References

1 Saunders, L. K.; Nowell, H.; Hatcher, L. E.; Shepherd, H. J.; Teat, S. J.; Allan, D. R.; Raithby, P. R.; Wilson, C. C. Exploring Short Strong Hydrogen Bonds Engineered in Organic Acid Molecular Crystals for Temperature Dependent Proton Migration Behaviour using Single Crystal Synchrotron X-ray Diffraction (SCSXRD), *CrystEngComm* **2019**, 21, 5249–5260.

2 Ravel, B.; Newville, M. Athena, Artemis, Hephaestus: Data Analysis for X-ray Absorption Spectroscopy using IFEFFIT, *J. Synchrotron Radiat.* **2005**, 12, 537–541.

3 Edwards, P. T.; Saunders, L. K.; Pallipurath, A. R.; Britton, A. J.; Willneff, E. A.; Shotton, E. J.; Schroeder, S. L. M. Proton Transfer on the Edge of the Salt/Cocrystal Continuum: X-Ray Photoelectron Spectroscopy of Three Isonicotinamide Salts, *Cryst. Growth Des.* **2021**, 21, 6334-6340.
